# High Number and Specific Comorbidities Could Impact the Immune Response in COVID-19 Patients

**DOI:** 10.3389/fimmu.2022.899930

**Published:** 2022-07-05

**Authors:** Dafeng Liu, Xiaoyan Yuan, Fengjiao Gao, Bennan Zhao, Ling Ding, Mingchang Huan, Chao Liu, Liangshuang Jiang

**Affiliations:** ^1^ Department of Internal Medicine, Public Health and Clinical Centre of Chengdu, Chengdu, China; ^2^ Public Health and Clinical Centre of Chengdu Substation, Chengdu New Emergent Infectious Disease Prevention and Control Workstation, Chengdu, China; ^3^ Department of Pediatrics, Public Health and Clinical Centre of Chengdu, Chengdu, China; ^4^ Department of Surgery, Public Health and Clinical Centre of Chengdu, Chengdu, China; ^5^ Vice President’s Office, Public Health and Clinical Centre of Chengdu, Chengdu, China

**Keywords:** lymphocyte subsets, coronavirus disease 2019 (COVID-19), comorbidities, impact, immune response

## Abstract

**Background:**

Cellular immunodeficiency and comorbidities are common in COVID-19 patients.

**Aim:**

The purpose of this study was to investigate comorbidities impacting on the cellular immunity in COVID-19 patients.

**Methods:**

The research objects included 55 healthy controls and 718 COVID-19 patients who divided into the control group and the COVID-19 group, respectively. Those in the COVID-19 group were divided into subgroups on the basis of the number and types of comorbidities present. Lymphocyte itself and its subsets were compared between the control group and the COVID-19 group, the groups with comorbidities based on the different number and types of comorbidities, and the relationship between the lymphocyte counts and subsets with the number and types of comorbidities was investigated.

**Results:**

Compared with the control group, the lymphocyte counts and T cell subsets were significantly increased in the groups with comorbidities, but both B and NK cell subsets were significantly decreased in the no comorbidity group and in most of the groups with comorbidities (all P<0.05). In the three comorbidities group, the lymphocyte counts and T cell subsets were all significantly decreased, but the CD56+ percentage was obviously increased (all P<0.05). The number of comorbidities was negatively correlated with the lymphocyte counts and the T and NK cell subsets. A negative correlation also existed between cancer and both the lymphocyte counts and the T cell subsets, between chronic hepatitis B and the lymphocyte counts, and between chronic kidney disease and the CD3+ counts. A positive correlation existed between nonalcoholic fatty liver disease (NAFLD) disease and both lymphocyte and CD3+ counts. The risk factors were number of comorbidities for the lymphocyte count, CD3+CD4+ and CD3+CD8+ percentages, NAFLD for the lymphocyte and CD3+ counts, cardiovascular diseases for CD3+CD4+ and CD3+CD8+ percentages, diabetes mellitus for the CD3+CD8+ percentage, and cancer for the CD3+ percentage, respectively.

**Conclusions:**

High numbers of comorbidities and specific comorbidities could impact the immune response of COVID-19 patients. This study provides a reference for clinicians in the identification of suitable and timely immunotherapy for COVID-19 patients.

**Clinical Trial Registry:**

https://www.chictr.org.cn/enindex.aspx, identifier ChiCTR2000034563.

## Introduction

Coronavirus disease 2019 (COVID-19), caused by infection with the severe acute respiratory syndrome coronavirus 2 virus (SARS-CoV-2), presentsan urgent threat and a paramount to global health and has caused a worldwide pandemic (1–5). As of March25, 2022, there were 476,374,234 confirmed cases, of them6,108,976cases died worldwide (6). COVID-19generally had a good prognosis (1–5), but a poor prognosis was found in those of old age, high number and specific comorbidities, and a rapid disease progression (7–12).The majority of COVID-19 patients who have died had pre-existing conditions, including hypertension, cardiovascular disease, diabetes, and cancer (10, 13).

As an important part of the dysfunction of regulating host immune response, what played an important role in the pathophysiology of COVID-19 (8, 14–18), SARS (19), and MERS-CoV (20)was the decline in cellular immune function. Host both innate and adaptive immune responses were altered due to SARS-COV-2 infection (21). The pathogenesis of hypertension and diabetes mellitus may potentially involve innate and adaptive immune responses (22–24). Growing evidence shows that adaptive (T and B lymphocytes, as well as monocytes/macrophages and dendritic cells) and innate (γ/δ T cells and natural killer cells) immune responses may be involved in the pathogenesis of hypertension (22). Systemic chronic low-grade inflammation is present in type 2 diabetes mellitus (T2DM). Function alteration of specific T lymphocyte subsets (including regulatory T (Treg) cells) results in the hypothesis that T2DM autoimmunity was exacerbated by partial inflammation (25–30). Our previous study showed that the lowest lymphocyte counts, especially T and B subsets were found in patients with severe COVID-19 and DM (15–18).

Few studies have reported on the impact of comorbidities on cellular immune responses in COVID-19 patients. Whether comorbidities (and which comorbidities are involved) can impact on the host immune response of patients with COVID-19 is unclear and worthy of studying.

## Methods

### Subjects

A cross-sectional research was used in this study.

**Figure 1:**
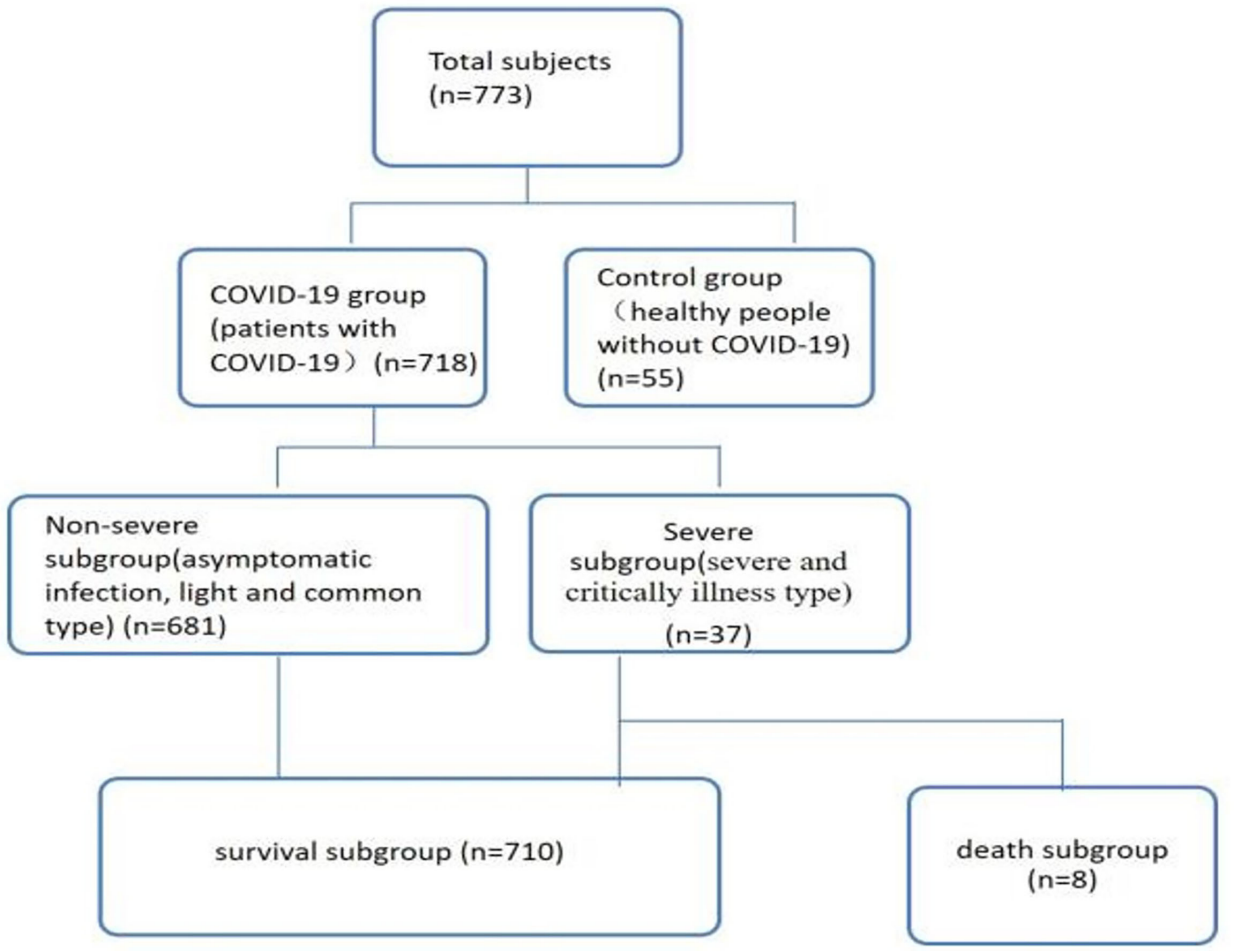
Patient data (*n*=773). Non-severe refers to the clinical type of COVID-19 that is asymptomatic, light and common. Severe refers to the clinical type of COVID-19 that is associated with severe and critical illness.

A total of 783 subjects, including 55 healthy controls who were from the medical examination clinic (16) and 718 COVID-19 patients who were from the first and second hospital isolation wards (15–18), and these patients were presented at Public Health and Clinical Center of Chengdu from 16 January 2020 to 30 April 2021 (**Figure 1**). The study was approved by the Ethics Committee of Public Health and Clinical Center of Chengdu (No.: PJ-K2020-26-01). Because this study evaluated an emerging infectious disease, the Ethics Commission of the designated hospital waived written informed consent.

### Diagnostic Criteria, Clinical Classification Criteria and Cure Criteria

Diagnostic criteria, clinical classification criteria and cure criteria for COVID-19 referred to the seventh Trial Version of the Novel Coronavirus Pneumonia Diagnosis and Treatment Guidance (7).

### Grouping Standards

The research objects included 55 healthy controls and 718 COVID-19 patients who divided into the control group and the COVID-19 group, respectively.

**Figure 2:**
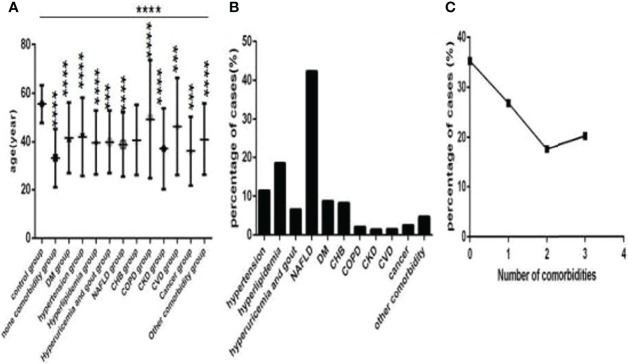
Comparison of age among the control group, the no comorbidity group and each comorbidity group, and the distribution characteristics of the number and type of comorbidities in COVID-19 patients (*n*=773). COVID-19, coronavirus disease 2019. NAFLD, nonalcoholic fatty liver disease. DM, diabetes mellitus. CHB, chronic hepatitis **(B)** COPD, chronic obstructive pulmonary disease. CKD, chronic kidney disease. CVD, cardiovascular disease. **(A)** age. **(B)** the percentage of each type of comorbidity. **(C)** The percentage of different numbers of comorbidities. One-way ANOVA was used for intergroup comparisons (P < 0.0001). Unpaired t-tests were used for comparisons with the control group, ***P < 0.001, ****P < 0.0001.

Of the 718 COVID-19 patients, 82 patients were in the hypertension subgroup (those with hypertension), 133 patients were in the hyperlipidemia subgroup (those with hyperlipidemia), 47 patients were in the hyperuricemia and gout subgroup (those with hyperuricemia and gout), 195 patients were in the nonalcoholic fatty liver disease (NAFLD) subgroup (those with non-alcoholic fatty liver disease), 63 patients were in the diabetes mellitus (DM) subgroup (those with DM), 59 patients were in the chronic hepatitis B (CHB) subgroup (those with CHB), 15 patients were in the chronic obstructive pulmonary disease (COPD) subgroup (those with COPD), 10 patients were in the chronic kidney disease (CKD) subgroup (those with CKD), 11 patients were in the cardiovascular disease (CVD) subgroup (those with CVD), 18 patients were in the cancer subgroup (those with cancer), and34 patients had other comorbidities, respectively (**Figure 2B**).

Among these patients, 253 patients were in the no comorbidity subgroup (patients without comorbidities), 193 patients were in the one comorbidity subgroup (patients with one type of comorbidity), 127 patients were in the two comorbidities subgroup (patients with two types of comorbidities), and 145 patients were in the three or more comorbidities subgroup (patients with three and more types of comorbidities) (**Figure 2C**), respectively.

Of the 718 COVID-19 patients, 39 patients were in the NAFLD plus DM subgroup (those with NAFLD and DM), 50 patients were in the NAFLD plus hypertension subgroup (those with NAFLD and hypertension), 88 patients were in the NAFLD plus hyperlipidemia subgroup (those with NAFLD and hyperlipidemia), 26 patients were in the NAFLD plus hyperuricemia and gout subgroup (those with NAFLD and hyperuricemia or gout), 13 patients were in the NAFLD plus CHB subgroup (those with NAFLD and CHB), 6 patients were in the NAFLD plus COPD subgroup (those with NAFLD and COPD), 6 patients were in the NAFLD plus CVD subgroup (those with NAFLD and CVD), 6 patients were in the NAFLD plus cancer subgroup (those with NAFLD and cancer), 17 patients were in the NAFLD plus DM plus hyperlipidemia subgroup (those with NAFLD, DM and hyperlipidemia), 19 patients were in the NAFLD plus hypertension plus hyperlipidemia subgroup (those with NAFLD, hypertension and hyperlipidemia), 11 patients were in the NAFLD plus hyperlipidemia plus hyperuricemia and gout subgroup (those with NAFLD, hyperlipidemia and hyperuricemia or gout), 14 patients were in the NAFLD plus DM plus hypertension subgroup (those with NAFLD, DM and hypertension), 5 patients were in the NAFLD plus DM plus hypertension plus hyperlipidemia subgroup (those with NAFLD, DM, hypertension and hyperlipidemia), 16 patients in the hyperlipidemia plus hyperuricemia and gout subgroup (those with hyperlipidemia and hyperuricemia or gout), 10 patients in the hyperlipidemia plus CHB subgroup (those with hyperlipidemia and CHB), 23 patients in the hyperlipidemia plus hyperuricemia and gout subgroup (those with hyperlipidemia and hypertension), 6 patients in the hypertension plus hyperuricemia and gout subgroup (those with hypertension and hyperuricemia or gout), 19 patients in the hypertension plus DM subgroup (those with hypertension and DM), 8 patients in the hypertension plus CHB subgroup (those with hypertension and CHB), 5 patients in the hypertension plus COPD subgroup (those with hypertension and COPD), 9 patients in the hypertension plus CKD subgroup (those with hypertension and CKD), 7 patients in the hypertension plus CVD subgroup (those with hypertension and CVD), 5 patients in the hypertension plus cancer subgroup (those with hypertension and cancer), 23 patients in the DM plus hyperlipidemia subgroup (those with DM and hyperlipidemia), 6 patients in the DM plus hyperuricemia and gout subgroup (those with DM and hyperuricemia or gout), 5 patients in the DM plus CHB subgroup (those with DM and CHB), 5 patients in the DM plus CKD subgroup (those with DM and CKD), 5 patients in the DM plus CVD subgroup (those with DM and CVD), respectively.

### Measurement of T-Lymphocyte Subsets

EDTA anticoagulated peripheral blood as flow cytometry samples was drawn from the participants before initial treatment to determine lymphocyte and subsets. All samples were tested within 6 hours of being obtained. Briefly, CD3+/CD4+/CD8+ T-cell, CD19+ B-cell, and CD16+ CD56+ NK-cell counts (cells/μL) were measured by multiple-color flow cytometry with human monoclonal anti-CD3-fluorescein isothiocyanate (FITC), anti-CD4-phycoerythrin (PE), antiCD8-allophycocyanin (APC), anti-CD19-PE, and anti-CD56-PE antibodies (BD Multitest) according to the manufacturer’s instructions. The cells were analyzed on a BD FACS Canto II flow cytometry system (BD Biosciences) (15, 19).

### Data Collection

We collected and recorded all of data, including clinical data, demographic data, lymphocyte itself and its subset from 773 objects, and strictly ensured the accuracy, completeness and authenticity of the data, then established the database.

### Statistical Analysis

The statistical and cartographic software, including SPSS 26.0 (SPSS, Chicago, IL, USA) and GraphPad Prism 8 (GraphPad, CA, USA) were used for statistical analyses and cartograph production. The expression of the measurement data isas x ± SD, and the comparisons of the homogeneity of the variance in the normally distributed data among multigroup use ANOVA. Then the comparisons between any two groups sue the least significant difference (LSD) t-test when the comparisons among multigroup have significant statistical significance. The comparisons between any two groups use an independent-sample t-test. The expression of categorical data is as a percentage or proportion, and the comparisons of these data use the chi-square test. Two-factor and multifactor correlation analysis use Spearman correlation analysis and multiple stepwise regression, respectively. Statistical significance was considered *P*<0.05.

## Results

### Baseline Conditions

Baseline information of 718 COVID-19 patied was showed (**Table 1**). Controls were older than COVID-19 patients (**Figure 2A**), and thoseCOVID-19 patients with comorbidities were older than those without comorbidities (**Figure 2A**), especially those with CVD and COPD (**Figure 2A**), statistically significance were found except for those with CKD (all *P*<0.001). Hypertension, NAFLD, DM, hyperlipidemia, hyperuricemia and gout and CHB were common in COVID-19 patients (**Figure 2B**). There was 253 cases with no comorbidity, 193 cases with one comorbidity, 127 cases with two comorbidities, and 145 cases with three or more comorbidities (**Figure 2C**).

**Table 1:** Baseline information (*n*=718).

Variables	χ±SD or case (%)	Range
Age (years)	38.48±14.15	0.17~87
Male (case, %)	529(73.68)	
Duration (day)	1.74±1.20	1~30天
Virus negative conversion time (days)	15.48±11.18	2~53天
In-hospital time (days)	18.28±11.16	2~56天
Severity		
Nonsevere	681(94.85)	
Severe	37(5.15)	
Prognosis		
Survival (case, %)	710(98.89)	
Death (case, %)	8(1.11)	

**Figure 3:**
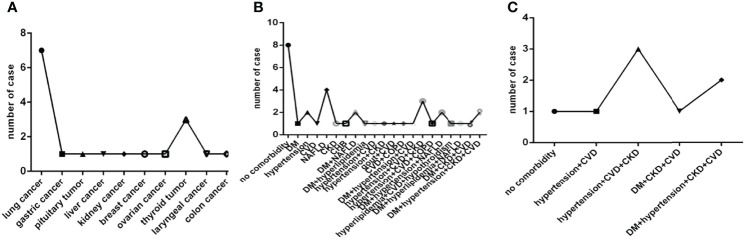
Comparison of the distribution characteristics of the type of cancer in eighteen patients with cancer, the distribution characteristics of the number and type of comorbidities in thirty-seven severe COVID-19 patients and in eight deadly COVID-19 patients. COVID-19, coronavirus disease 2019. NAFLD, nonalcoholic fatty liver disease. DM, diabetes mellitus. CHB, chronic hepatitis **(B)** COPD, chronic obstructive pulmonary disease. CKD, chronic kidney disease. CVD, cardiovascular disease. **(A)** The type of cancer. **(B)** The number and type of comorbidities in severe COVID-19 patients. **(C)** The number and type of comorbidities in deadly COVID-19 patients.

Of them eighteen patients had cancer, that was seven with lung cancer, three with thyroid cancer, one with gastric cancer, pituitary tumor, liver cancer, kidney cancer, breast cancer, ovarian cancer, laryngeal cancer, colon cancer, respectively. Only colon cancer was active, others were non-active (**Figure 3A**).

In thirty-seven severe cases there were eight cases without comorbidity, ten cases with one comorbidity, seven cases with two comorbidities and twelve cases with three or more comorbidities. Hypertension, NAFLD, DM, CVD, CKD, CHB, COPD and a combination of them were common in severe COVID-19 patients (**Figure 3B**). While in eight deadly cases there were only one case without comorbidity, one case with two comorbidities, six cases with three or more comorbidities, and only hypertension, DM, CVD, CKD and a combination of them were found in deadly cases (**Figure 3C**).

### Comparison of Baseline Lymphocytes and Subsets Among the Control Group, the No Comorbidity Group and the One Comorbidity Group

**Figure 4:**
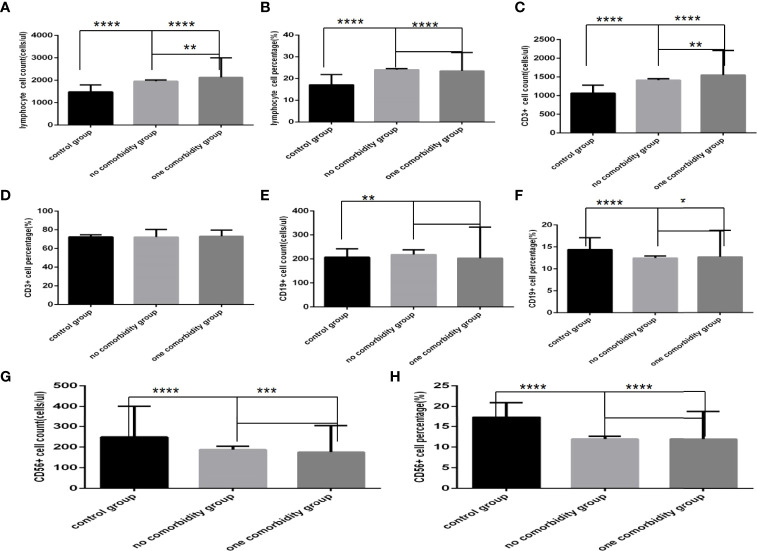
Comparison of lymphocyte, T, B and NK cell subset counts and percentages among the control group, the no comorbidity group and the one comorbidity group (*n*=494; control, no, one comorbidity groups, *n*=55, 246 and 193 cases, respectively). COVID-19, coronavirus disease 2019. NAFLD, nonalcoholic fatty liver disease. DM, diabetes mellitus. CHB, chronic hepatitis **(B)** COPD, chronic obstructive pulmonary disease. CKD, chronic kidney disease. CVD, cardiovascular disease. **(A)** lymphocyte count. **(B)** lymphocyte percentage. **(C)** CD3+ count. **(D)** CD3+ percentage. **(E)** CD19+ count. **(F)** CD19+ percentage. **(G)** CD56+ count. **(H)** CD56+ percentage. One-way ANOVA was used for intergroup comparisons (**A, B, C, G, H**, *P* all<0.0001; F, *P*<0.01; **D, E**, *P* all>0.05). Unpaired *t*-tests were used for comparisons with the control group, and with the no comorbidity group, *P < 0.05, **P < 0.01, ***P < 0.001, ****P < 0.0001.

**Figure 5:**
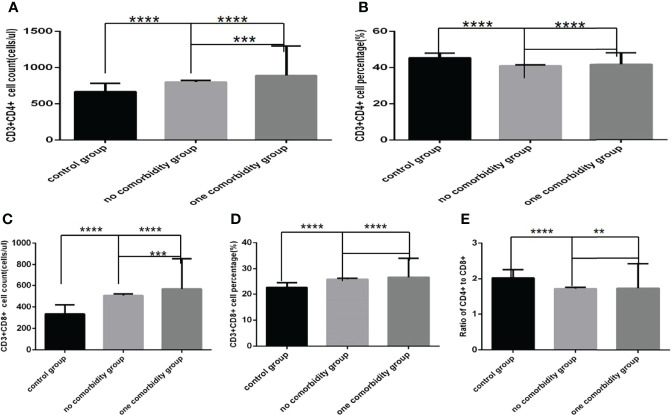
Comparison of T cell subset counts and percentages the control group, the no comorbidity group and the one comorbidity group (*n*=494; control, no, one comorbidity groups, *n*=55, 246 and 193 cases, respectively). COVID-19, coronavirus disease 2019. NAFLD, nonalcoholic fatty liver disease. DM, diabetes mellitus. CHB, chronic hepatitis **(B)** COPD, chronic obstructive pulmonary disease. CKD, chronic kidney disease. CVD, cardiovascular disease. **(A)** CD3+CD4+ cell count. **(B)** CD3+CD4+ cell percentage. **(C)** CD3+CD8+ cell count. **(D)** CD3+CD8+ cell percentage. **(E)** Ratio of CD4+/CD8+ cells. Unpaired one-way ANOVA was used for intergroup comparisons (**A, B, C, D, E**, *P* all<0.0001). Unpaired *t*-tests were used for comparisons with the control group, and with the no comorbidity group, **P < 0.01, ***P < 0.001, ****P < 0.0001.

Comparison with those in the control group, lymphocyte counts and percentage, CD3+ counts, CD3+CD4+ counts, CD3+CD8+ counts and percentage (**Figures 4A–C**, **5A–D**)were higher, while CD19+ counts and percentage, and CD56+ counts and percentage, CD3+CD4+ percentage, and the ratio of CD3+CD4+ to CD3+CD8+ (**Figures 4E–H**, **5B, E**) were significantly lower in the no comorbidity group; significant differences were found (*P*<0.0001, 0,0001, 0.0001, 0,0001,0.0001, 0,0001,0.01, 0,0001,0.0001, 0,0001,0.0001, 0,0001, respectively). But there was no difference of CD3+ percentage between these two groups (**Figure 4D**) (*P*>0.05).

Comparison with those in the control group, lymphocyte counts and percentage, CD3+ counts, CD3+CD4+ counts, CD3+CD8+ counts and percentage (**Figures 4A–C**, **5A–D**)were higher, while CD19+ percentage, and CD56+ counts and percentage, CD3+CD4+ percentage, and the ratio of CD3+CD4+ to CD3+CD8+ (**Figures 4F–H**, **5B, E**) were significantly lower in the one comorbidity group; significant differences were found (*P*<0.0001, 0.0001, 0.0001, 0.05,0.001, 0.0001,0.0001, 0.0001,0.0001, 0.0001,0.01, respectively). But there was no difference of CD3+ percentage and CD19+ counts between these two groups (**Figures 4D, E**) (*P*all *>*0.05).

Moreover, compared with the no comorbidity group, lymphocyte counts, CD3+ counts, CD3+CD4+ counts and CD3+CD8+ counts (**Figures 4A, C**, **5A, C**) were higher in the one comorbidity group; significant differences were found (*P*<0.01, 0.01, 0.001, 0.001, respectively). But there was no difference of lymphocyte percentage, CD3+percentage, CD19+ counts and percentage, and CD56+ counts and percentage, CD3+CD4+ percentage, CD3+CD8+ percentage, and the ratio of CD3+CD4+ to CD3+CD8+ between the no comorbidity group and the one comorbidity groups (**Figures 4B, D–H**, **5B, D, E**) (*P* all *>*0.05).

### Comparison of Baseline Lymphocytes and Subsets Among Different Number of Comorbidities Groups in COVID-19 Patients

**Figure 6:**
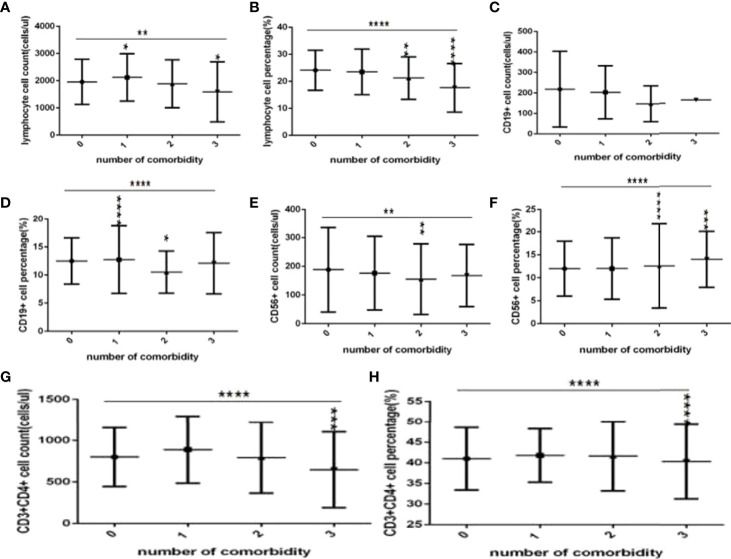
Comparison of lymphocyte, T, B and NK cell subset counts and percentages among the different numbers of comorbidities (*n*=718; 0, 1, 2, 3 or more comorbidity groups, *n*=253, 193, 127 and 145 cases, respectively). C0OVID-19, coronavirus disease 2019. NAFLD, nonalcoholic fatty liver disease. DM, diabetes mellitus. CHB, chronic hepatitis **(B)** COPD, chronic obstructive pulmonary disease. CKD, chronic kidney disease. CVD, cardiovascular disease. **(A)** lymphocyte count. **(B)** lymphocyte percentage. **(C)** CD19+ count. **(D)** CD19+ percentage. **(E)** CD56+ count. **(F)** CD56+ percentage. **(G)** CD3+ count. **(H)** CD3+ percentage. Unpaired one-way ANOVA was used for intergroup comparisons (**B, D, F, G, H** *P* all < 0.0001; **A, C**, *P* all <0.01; **E**, *P*>0.05). Unpaired *t*-tests were used for comparisons with the no comorbidity group, *P < 0.05, **P < 0.01, ***P < 0.001, ****P < 0.0001.

**Figure 7:**
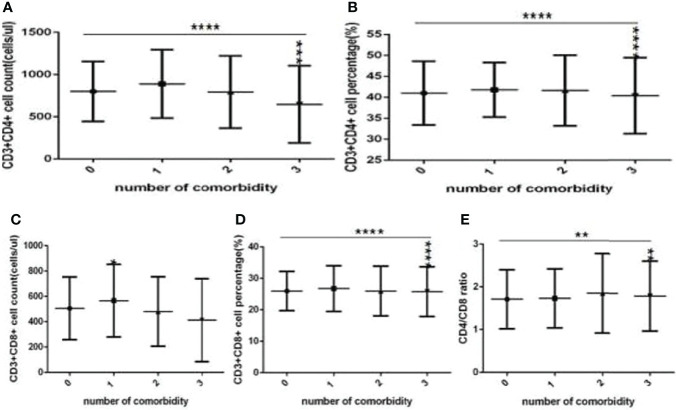
Comparison of T cell subset counts and percentages among the different numbers of comorbidities (*n*=718; 0, 1, 2, 3 or more comorbidity groups, *n*=253, 193, 127 and 145 cases, respectively). COVID-19, coronavirus disease 2019. NAFLD, nonalcoholic fatty liver disease. DM, diabetes mellitus. CHB, chronic hepatitis **(B)** COPD, chronic obstructive pulmonary disease. CKD, chronic kidney disease. CVD, cardiovascular disease. **(A)** CD3+CD4+ cell count. **(B)** CD3+CD4+ cell percentage. **(C)** CD3+CD8+ cell count. **(D)** CD3+CD8+ cell percentage. **(E)** Ratio of CD4+/CD8+ cells. Unpaired one-way ANOVA was used for intergroup comparisons (**A, B, D**, *P* all <0.0001; **E**, *P* < 0.01; **C**, *P* > 0.05). Unpaired *t*-tests were used for comparisons with the no comorbidity group, *P < 0.05, **P < 0.01, ***P < 0.001, ****P < 0.0001.

Comparison with those in the no comorbidity group, lymphocyte counts, CD3+ counts and CD3+CD8+ counts (**Figures 6A, G**, **7C**), were slightly higher in the one comorbidity group, the CD19+ percentage (**Figure 6D**) was significantly higher; significant differences were found (*P*<0.05, 0.05, 0.05, 0.0001, respectively). But there was no difference of lymphocyte percentage, CD3+ percentage, CD19+ counts, CD56+ counts and percentage, CD3+CD4+ counts and percentage, CD3+CD8+ percentage, and the ratio of CD3+CD4+ to CD3+CD8+ between these two groups (**Figures 6B, D, E, G, H**, **7A, B, D, E**) (*P* all >0.05).

Moreover, compared with the no comorbidity group, the lymphocyte percentages, CD19+ percentages and CD56+ counts (**Figures 6B, D, E**) were significantly lower in the two comorbidities group, while the CD56+ percentages (**Figure 6F**) were significantly higher in the two comorbidities group; these differences were significant (*P*<0.01, 0.05, 0.01, 0.0001, respectively). But there was no difference of lymphocyte counts, CD3+ counts and percentage, CD19+ counts, CD3+CD4+ counts and percentage, CD3+CD8+ counts and percentage, and the ratio of CD3+CD4+ to CD3+CD8+ between these two groups (**Figures 6A,C–E**, **7A–E**) (*P* all >0.05).

Furthermore, compared with the no comorbidity group, in the three or more comorbidities group, lymphocyte counts and percentage, the CD3+ counts and percentages, and CD3+CD4+ counts and percentages, and CD3+CD8+ counts (**Figures 6A, B, G, H**, **7A–C**) were all significantly lower, but the CD56+ percentage,CD3+CD8+ percentages, and the ratio of CD3+CD4+ to CD3+CD8+ (**Figures 6F**, **7D, E**) were obviously higher; these differences were significant (*P*<0.05, 0.0001, 0.0001, 0.05, 0.001, 0.0001, 0.0001, 0.001, 0.01, 0.01, respectively). But there was no difference of CD19+ counts and percentage, CD56+ counts, CD3+CD8+ percentage, and the ratio of CD3+CD4+ to CD3+CD8+ between these two groups (**Figures 6E–G**, **7D, E**) (*P* all >0.05).

### Comparisons of Baseline Lymphocytes and Subsets Among the Control Group, the No Comorbidity Group and the Different Types of Comorbidities Group in COVID-19 Patients

**Figure 8:**
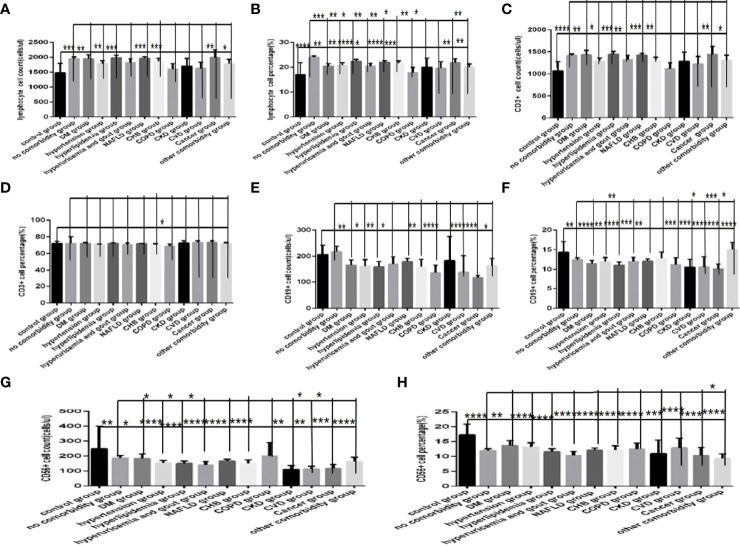
Comparison of lymphocyte, T, B and NK cell subset counts and percentages among the control group, the no comorbidity group and each type of comorbidity group (*n*=773; control, no comorbidity and each type of comorbidity group, *n*=55, 253, 82, 133, 47, 195, 63, 59, 15, 10, 11, 18 and 34 cases, respectively). Abbreviations: COVID-19, coronavirus disease 2019. NAFLD, nonalcoholic fatty liver disease. DM, diabetes mellitus. CHB, chronic hepatitis **(B)** COPD, chronic obstructive pulmonary disease. CKD, chronic kidney disease. CVD, cardiovascular disease. **(A)** lymphocyte count. **(B)** lymphocyte percentage. **(C)** CD3+ count. **(D)** CD3+ percentage. **(E)** CD19+ count. **(F)** CD19+ percentage. **(G)** CD56+ count. **(H)** CD56+ percentage. Unpaired one-way ANOVA was used for intergroup comparisons (**B, E, F, G, H**, *P* all < 0.0001; **A, C**, *P* all < 0.05; **D**, *P* > 0.05). Unpaired *t*-tests were used for comparisons with the control group, and with the no comorbidity group, *P < 0.05, **P < 0.01, ***P < 0.001, ****P<0.0001.

**Figure 9:**
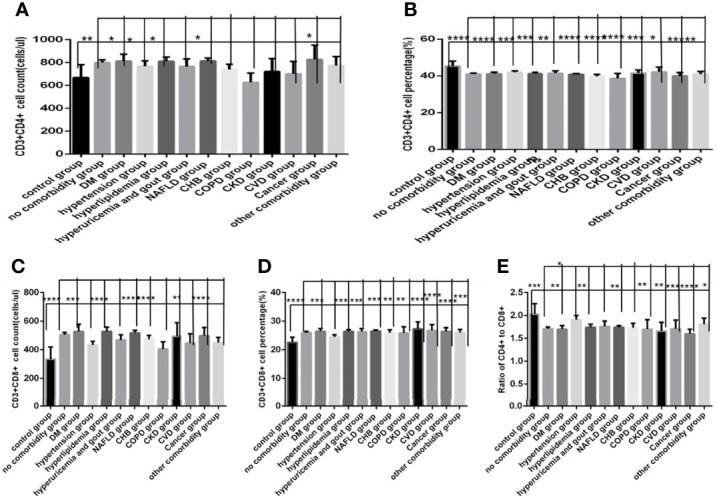
Comparison of T lymphocyte subset counts and percentages among the control group, the no comorbidity group and each type of comorbidity group (*n*=773; control, no comorbidity and each type of comorbidity group, *n*=55, 253, 82, 133, 47, 195, 63, 59, 15, 10, 11, 18 and 34 cases, respectively). Abbreviations: COVID-19, coronavirus disease 2019. NAFLD, nonalcoholic fatty liver disease. DM, diabetes mellitus. CHB, chronic hepatitis **(B)** COPD, chronic obstructive pulmonary disease. CKD, chronic kidney disease. CVD, cardiovascular disease. **(A)** CD3+CD4+ cell count. **(B)** CD3+CD4+ cell percentage. **(C)** CD3+CD8+ cell count. **(D)** CD3+CD8+ cell percentage. **(E)** Ratio of CD4+/CD8+ cells. Unpaired one-way ANOVA was used for intergroup comparisons (**C**, *P* < 0.01; **B**, **D**, *P* all < 0.05; A, *P* > 0.05). Unpaired *t*-tests were used for comparisons with the control group, and with the no comorbidity group, *P < 0.05, **P < 0.01, ***P < 0.001, ****P < 0.0001.

Comparison with those in the control group, the lymphocytes counts and percentages, CD3+ counts, CD3+CD4+ counts,CD3+CD8+ counts and percentages (**Figures 8A–C**, **9A, C, D**), were significantly increased, while the CD19+counts and percentages, CD56+ counts and percentages, CD3+CD4+ percentages and the ratios of CD3+CD4+ to CD3+CD8+ (**Figures 8D–H**, **9B, E**) were significantly decreased in the DM group, the hypertension group, the hyperlipidemia group, the NAFLD group, the CHB group, the cancer group and the other comorbidity group (*P* all <0.05).But there was no difference of CD3+ percentage between the control group and the DM group, the hypertension group, the hyperlipidemia group, the NAFLD group, the CHB group, the cancer group, or the other comorbidity group (**Figure 8C**) (*P* all >0.05).

Moreover, comparison with those in the no comorbidity group, in most of the subtype groups for different comorbidities, the lymphocyte percentages, CD19+ percentages and CD56+ counts (**Figures 8B, F, G**) were significantly decreased (*P*all *<*0.05).

### Comparisons of Baseline Lymphocytes and Subsets Between the Specific Comorbidity Group and the Different Combination of Specific Comorbidity With Other Comorbidities Group in COVID-19 Patients

**Figure 10:**
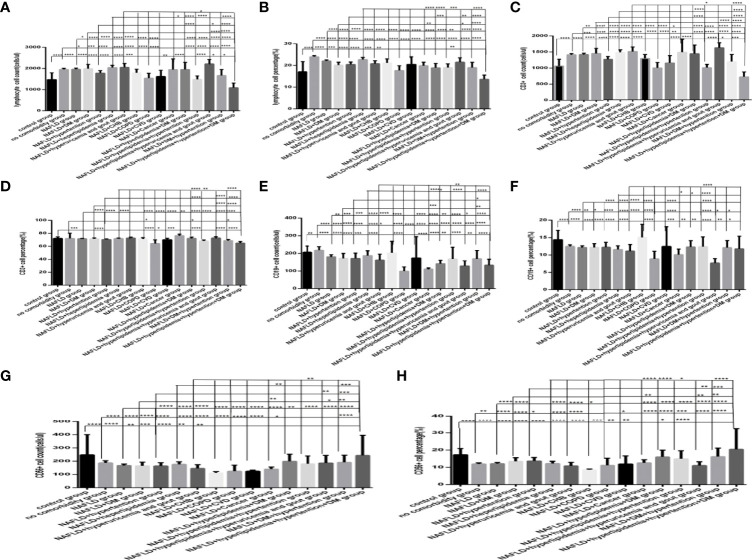
Comparison of lymphocyte, T, B and NK cell subset counts and percentages among the control group, the no comorbidity group, with NAFLD group and with each combination of NAFLD and other comorbidities group (*n*=773; control, no comorbidity, NAFLD, and each combination of NAFLD and other comorbidities group, *n*=55, 253, 304, 39, 50, 88, 26, 13, 6, 6, 6, 17, 19, 11, 14 and 5 cases, respectively). Abbreviations: CHB, chronic hepatitis B.CKD, chronic kidney disease. COPD, chronic obstructive pulmonary disease. COVID-19, coronavirus disease 2019.CVD, cardiovascular disease. NAFLD, nonalcoholic fatty liver disease. DM, diabetes mellitus. HT, Hypertention. HL, heperlipidemia. HU, hyperuricemia and gout. **(A)** lymphocyte count. **(B)** lymphocyte percentage. **(C)** CD3+ count. **(D)** CD3+ percentage. **(E)** CD19+ count. **(F)** CD19+ percentage. **(G)** CD56+ count. **(H)** CD56+ percentage. Unpaired one-way ANOVA was used for intergroup comparisons (**A, B, C, D, E, F, G, H**, *P* all < 0.0001). Unpaired *t*-tests were used for comparisons with the control group, and with the no comorbidity group, with NAFLD group, with NAFLD plus DM group, with NAFLD plus hypertension group, with NAFLD plus hyperlipidemia group, with NAFLD plus hyperuricemia and gout group, *P < 0.05, **P < 0.01, ***P < 0.001, ****P < 0.0001.

**Figure 11:**
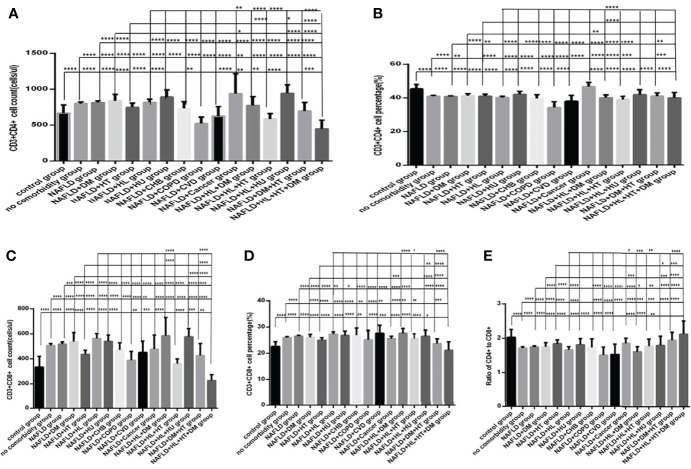
Comparison of T lymphocyte subset counts and percentages among the control group, the no comorbidity group, with NAFLD group and with each combination of NAFLD and other comorbidities group (*n*=773; control, no comorbidity, NAFLD, and each combination of NAFLD and other comorbidities group, *n*=55, 253, 304, 39, 50, 88, 26, 13, 6, 6, 6, 17, 19, 11, 14 and 5 cases, respectively). CHB, chronic hepatitis B.CKD, chronic kidney disease. COPD, chronic obstructive pulmonary disease. COVID-19, coronavirus disease 2019.CVD, cardiovascular disease. NAFLD, nonalcoholic fatty liver disease. DM, diabetes mellitus. HT, Hypertention. HL, heperlipidemia. HU, hyperuricemia and gout. **(A)** CD3+CD4+ cell count. **(B)** CD3+CD4+ cell percentage. **(C)** CD3+CD8+ cell count. **(D)** CD3+CD8+ cell percentage. **(E)** Ratio of CD4+/CD8+ cells. Unpaired one-way ANOVA was used for intergroup comparisons (**A–E**, *P* all < 0.0001). Unpaired *t*-tests were used for comparisons with the control group, and with the no comorbidity group, with NAFLD group, with NAFLD plus DM group, with NAFLD plus hypertension group, with NAFLD plus hyperlipidemia group, with NAFLD plus hyperuricemia and gout group, *P < 0.05, **P < 0.01, ***P < 0.001, ****P < 0.0001.

Comparison with those in the NAFLD group, the lymphocytes counts and percentages, CD3+ counts and percentages, CD19+ counts and percentages, CD56+ counts and percentages, CD3+CD4+ counts and percentages, CD3+CD8+ counts and percentages, and the ratios of CD3+CD4+ to CD3+CD8+ (**Figures 10A–H**, **11A–E**), were significantly increased in the NAFLD plus DM group, in the NAFLD plus hyperlipidemia group, in the NAFLD plus hyperuricemia and gout group; while those were significantly decreased in the NAFLD plus hypertension group, in the NAFLD plus CHB group, in the NAFLD plus COPD group, in the NAFLD plus CVD group, in the NAFLD plus CKD group, and in the NAFLD plus cancer group (*P* all <0.05).

Moreover, comparison with those in the NAFLD plus one specific comorbidity group, the lymphocytes counts and percentages, CD3+ counts and percentages, CD19+ counts and percentages, CD56+ counts and percentages, CD3+CD4+ counts and percentages, CD3+CD8+ counts and percentages, and the ratios of CD3+CD4+ to CD3+CD8+ (**Figures 10A–H**, **11A–E**), were significantly decreased in the NAFLD plus two or more specific comorbidity group (*P* all <0.05).

**Figure 12:**
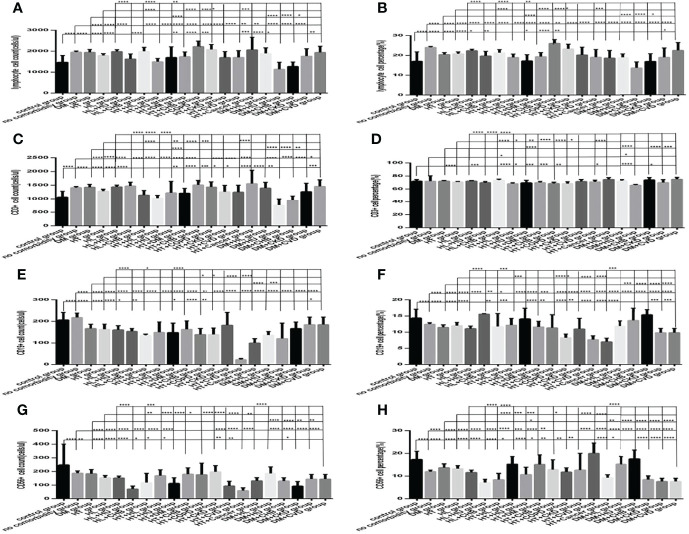
Comparison of lymphocyte, T, B and NK cell subset counts and percentages among the control group, the no comorbidity group, with DM group, with hypertension group, with hyperlipidemia group, and with each combination of DM, or hypertension, or hyperlipidemia and other comorbidities group (*n*=773; control, no comorbidity, DM, hypertension, hyperlipidemia and each combination of DM, or hypertension, or hyperlipidemia and other comorbidities group, *n*=55, 253, 63, 82, 133, 16, 10, 23, 6, 19, 8, 5, 9, 7, 5, 23, 6, 5, 5 and 5 cases, respectively). CHB, chronic hepatitis B.CKD, chronic kidney disease. COPD, chronic obstructive pulmonary disease. COVID-19, coronavirus disease 2019.CVD, cardiovascular disease. NAFLD, nonalcoholic fatty liver disease. DM, diabetes mellitus. HT, Hypertention. HL, heperlipidemia. HU, hyperuricemia and gout. **(A)** lymphocyte count. **(B)** lymphocyte percentage. **(C)** CD3+ count. **(D)** CD3+ percentage. **(E)** CD19+ count. **(F)** CD19+ percentage. **(G)** CD56+ count. **(H)** CD56+ percentage. Unpaired one-way ANOVA was used for intergroup comparisons (**A–C, E–H**, *P* all < 0.0001; **D**, *P* all < 0.001). Unpaired *t*-tests were used for comparisons with the control group, and with the no comorbidity group, with DM group, with hypertension group, with hyperlipidemia group, *P < 0.05, **P < 0.01, ***P < 0.001, ****P < 0.0001.

**Figure 13:**
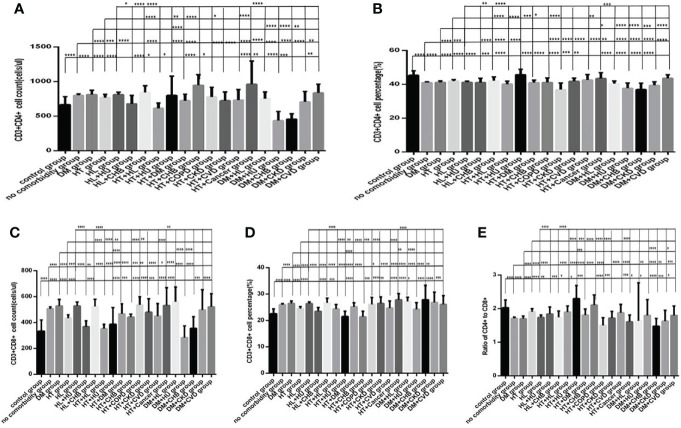
Comparison of T lymphocyte subset counts and percentages among the control group, the no comorbidity group, with DM group, with hypertension group, with hyperlipidemia group, and with each combination of DM, or hypertension, or hyperlipidemia and other comorbidities group(*n*=773; control, no comorbidity, DM, hypertension, hyperlipidemia and each combination of DM, or hypertension, or hyperlipidemia and other comorbidities group, *n*=55, 253, 63, 82, 133, 16, 10, 23, 6, 19, 8, 5, 9, 7, 5, 23, 6, 5, 5 and 5 cases, respectively). CHB, chronic hepatitis B.CKD, chronic kidney disease. COPD, chronic obstructive pulmonary disease. COVID-19, coronavirus disease 2019.CVD, cardiovascular disease. NAFLD, nonalcoholic fatty liver disease. DM, diabetes mellitus. HT, Hypertention. HL, heperlipidemia. HU, hyperuricemia and gout. **(A)** CD3+CD4+ cell count. **(B)** CD3+CD4+ cell percentage. **(C)** CD3+CD8+ cell count. **(D)** CD3+CD8+ cell percentage. **(E)** Ratio of CD4+/CD8+ cells. Unpaired one-way ANOVA was used for intergroup comparisons (**A–E**, *P* all < 0.0001). Unpaired *t*-tests were used for comparisons with the control group, and with the no comorbidity group, with DM group, with hypertension group, with hyperlipidemia group, *P < 0.05, **P < 0.01, ***P < 0.001, ****P < 0.0001.

Comparison with those in the one specific comorbidity such as DM, hypertension, hyperlipidemia group, the lymphocytes counts and percentages, CD3+ counts and percentages, CD19+ counts and percentages, CD56+ counts and percentages, CD3+CD4+ counts and percentages, CD3+CD8+ counts and percentages, and the ratios of CD3+CD4+ to CD3+CD8+ (**Figures 12A–H**, **13A–E**), were significantly reduced in the specific comorbidity plus another comorbidity group, in the specific comorbidity plus two or more other comorbidities group (*P* all <0.05).

### The Relationship of the Number and Type of Comorbidities With Lymphocytes and Subsets in COVID-19 Patients

By Spearman correlation analysis, lymphocytes counts and percentages, T cell subset counts and percentages, including CD3+ counts and percentages, CD3+CD4+ counts and percentages, and the ratios of CD3+CD4+ to CD3+CD8+, and NK cell subset counts and percentages were all negatively correlated with the amounts of comorbidities. Lymphocyte count and percentages, T cell subset counts and percentages, including CD3+CD8+ counts, CD3+CD4+ counts and percentages, CD3+ counts and percentages, and the ratios of CD3+CD4+ to CD3+CD8+ were also negatively correlated, but CD3+CD8+ percentage was negatively correlated with Cancer. Lymphocyte count and percentages, T cell subset counts and percentages, including CD3+CD8+ counts, CD3+CD4+ counts and percentages, and the ratios of CD3+CD4+ to CD3+CD8+ were also negatively correlated, but CD3+CD8+ percentage was also positively correlated with CHB. Only CD3+ count was negatively correlated with CKD. T cell subset counts and percentages, including the CD3+CD4+ percentage, CD3+CD8+ percentages, and the ratios of CD3+CD4+ to CD3+CD8+were negatively correlated, but CD3+CD8+ counts was positively correlated with CVD. And lymphocyte counts and CD3+ counts was positively correlated with NAFLD (**Table 2**–**4**).

**Table 2:** Spearman and partial correlation analysis of lymphocytes, age, sex, duration, geographical source of infection and comorbidities (n = 718).

Control Variable	Variable	LY (cells/µl)		LY%	
		r	p	r	p
	Age (years)	-0.234	<0.0001	-0.286	<0.0001
	Number of comorbidities	-0.290	<0.0001	-0.350	<0.0001
	Nonalcoholic fatty liver disease (1=without, 2=with)	0.114	0.010		
	Chronic hepatitis B (1=without, 2=with)	-0.155	<0.0001	-0.157	<0.0001
	CancerCancer (1=without, 2=with)	-0.204	<0.0001	-0.180	<0.0001
	Other comorbidities (1=without, 2=with)			-0.109	0.014
age	Number of comorbidities			-0.195	0.003

LY, lymphocytes.

**Table 3:** Spearman correlation analysis of lymphocyte subsets, age, sex, duration, geographical source of infection and comorbidities (n = 718).

variable	CD3+ (cells/µl)		CD3%		CD56+ (cells/µl)		CD56+ %	
	r	p	r	p	r	p	r	p
Age (years)	-0.245	<0.0001						
Number of comorbidities	-0.293	<0.0001	-0.100	0.007	-0.181	0.005	-0.141	0.029
Nonalcoholic fatty liver disease (1=without, 2=with)	0.088	0.047						
Chronic kidney disease (1=without, 2=with)	-0.177	<0.0001						
Cancer (1=without, 2=with)	-0.227	<0.0001	0.100	0.024				

LY, lymphocytes.

**Table 4:** Spearman correlation analysis of T cell subset, age, sex, duration, geographical source of infection and comorbidities (n=718).

variable	CD3+CD4+ (cells/µl)		CD3+CD4+%		CD3+CD8+ (cells/µl)		CD3+CD8+%		CD4/CD8	
	r	p	r	p	r	p	r	p	r	p
Age (years)	-0.173	<0.0001	-0.103	0.020	-0.262	<0.0001	-0.090	0.043	0.121	0.006
Number of comorbidities	-0.186	<0.0001	-0.141	0.001					-0.137	0.002
Diabetes mellitus (1=without, 2=with)					0.090	0.043				
Nonalcoholic fatty liver disease (1=without, 2=with)	0.088	0.047			0.120	0.006				
Chronic hepatitis B (1=without, 2=with)	-0.177	<0.0001	-0.119	0.007	-0.113	0.010	0.089	0.044	-0.120	0.007
Cardiovascular diseases (1=without, 2=with)			-0.119	0.007	0.117	0.008	-0.121	0.006	-0.117	0.008
Cancer (1=without, 2=with)	-0.277	<0.0001	-0.148	0.001	-0.118	0.007	0.209	<0.0001	-0.198	0.000

LY, lymphocytes.

When controlling for age during the partial correlation analysis, there was only one negative correlation between lymphocyte counts and the amounts of comorbidities existed, and the other correlations were no longer present (**Table 2**–**4**).

Moreover, by multiple stepwise regression analysis, the amounts of comorbidities were the risk factor for lymphocyte percentages, CD3+CD4+ percentage, CD3+CD8+ percentage, nonalcoholic fatty liver disease was the risk factor for lymphocyte counts and CD3+ counts, cardiovascular disease was the risk factor for CD3+CD4+ percentage and CD3+CD8+ percentage, diabetes mellitus was the risk factor for CD3+CD8+ percentage, and cancer was the risk factor forCD3+ percentage (**Table 5**).

**Table 5:** Multiple stepwise regression analysis of comorbidities influencing lymphocyte and subsets (n = 718) .

independent variable		B	Std. Error	Beta	t	p
LY (cells/µl)	constant	2018.110	56.539	–	35.694	<0.0001
	Number of comorbidities (0, 1, 2, 3 and more)	-130.946	30.436	-0.161	-4.302	<0.0001
	Nonalcoholic fatty liver disease (1=without, 2=with)	139.570	70.560	0.074	1.978	0.048
LY%	constant	24.680	0.454	–	54.396	<0.0001
	Number of comorbidities (0, 1, 2, 3 and more)	-2.102	0.266	-0.283	-7.895	<0.0001
CD3+(cells/µl)	constant	1463.769	41.189	–	35.538	<0.0001
	Number of comorbidities (0, 1, 2, 3 and more)	-102.201	22.173	-0.172	-4.609	<0.0001
	Nonalcoholic fatty liver disease (1=without, 2=with)	108.320	51.404	0.079	2.107	0.035
CD3+%	constant	72.996	0.404	–	180.757	<0.0001
	Number of comorbidities (0, 1, 2, 3 and more)	-0.679	0.237	-0.107	-2.864	0.004
CD19+ (cells/µl)	constant	216.741	18.010	–	12.034	<0.0001
	Number of comorbidities (0, 1, 2, 3 and more)	-21.507	10.113	-0.137	-2.127	0.034
CD3+CD4+(cells/µl)	constant	852.535	22.772	–	37.438	<0.0001
	Number of comorbidities (0, 1, 2, 3 and more)	-49.001	13.364	-0.136	-3.667	<0.0001
CD3+CD8+(cells/µl)	constant	527.267	17.446	–	30.224	<0.0001
	Number of comorbidities (0, 1, 2, 3 and more)	-34.540	9.348	-0.138	-3.696	<0.0001
	Nonalcoholic fatty liver disease (1=without, 2=with)	53.488	21.798	0.092	2.404	0.014
	hypertension (1=without, 2=with)	-73.162	33.520	-0.081	-2.183	0.029
CD3+CD8+%	constant	26.344	0.285	–	92.512	<0.0001
	hypertension (1=without, 2=with)	-1.870	0.841	-0.083	-2.223	0.027

LY, lymphocytes.

## Discussion

In this study, we found that comparison with those in the control group, lymphocyte itself and T cell subset were significantly increased in the no comorbidity group and in the one comorbidity group, B and NK cell subset were significantly decreased in the no comorbidity group and slightly decreased in the one comorbidity group. But comparison with those in the no comorbidity group, in the two comorbidities group and in the three or more comorbidities group the lymphocyte itself and T, B, and NK cell subsets were all decreased, especially NK cell subset in the two comorbidities group and lymphocyte itself and T cell subset in the three or more comorbidities group. Having more comorbidities was negatively correlated with the lymphocyte counts and the T and NK cell subsets and was also risk factors for the lymphocyte counts and the CD3+CD4+ and CD3+CD8+ percentages. Previous studies have shown that having a high number of comorbidities is a risk factor for worse outcomes (19, 20, 25–28), and our previous study showed that a high number of comorbidities had predictive value in differentiating non-severe patients from severe patients and in distinguishing patients who died versus patients who survived. However, there are no reports in the literature regarding the impact of the number of comorbidities on host immune response of patients with COVID-19. This study was the first to find that the number of comorbidities can impact the immune response in COVID-19 patients, and it has been shown that having 2 or more comorbidities is associated with a decrease in the lymphocyte count and the T and NK cell subsets in COVID-19 patients.

This study findings showed that compared with the no comorbidity group, in most of the comorbidity subgroups, the lymphocyte percentages and CD19+ and CD56+ counts were significantly decreased. Comparison with those in the no comorbidity group, only NK cell subset was reduced in some specific comorbidity group, such as hypertension, hyperlipidemia, hyperuricemia and gout, CVD and cancer. Moreover, comparison with those in the NAFLD group, lymphocyte itself and T, B, NK cell subsets were all significantly increased in the NAFLD plus some specific comorbidity group such as DM, hyperlipidemia and hyperuricemia and gout; while those were significantly decreased in the NAFLD plus other specific comorbidity group such as hypertension, CHB, COPD, CVD, CKD and cancer. The same changes were seen between in the DM group, in the hypertension group, in the hyperlipidemia group and in the one of them plus some specific comorbidity group. The more comorbidities combination, the greater the reduction of lymphocyte itself and T, B, NK cell subsets. Furthermore, there were negative correlations between cancer and the lymphocyte counts and T cell subset, between CHB and the lymphocyte counts and T cell subset (especially CD3+CD4+),and between CKD and the CD3+ counts. There was a positive correlation between NAFLD and the lymphocyte and CD3+ counts. Furthermore, NAFLD for lymphocyte and CD3+ counts, CVD for CD3+CD4+ and CD3+CD8+ percentage, DM for CD3+CD8+ percentage, and cancer for CD3+ percentage were all shown to be risk factors. These findings show that specific comorbidities, including cancer, CHB, CKD, NAFLD, CVD and DM, could impact the immune response of COVID-19 patients. A previous study reported that in CHB, impaired helper CD4+ T cells was considered to be a central component of overall HBV-specific immune dysfunction. HBV-specific CD4+ T cell response reducing would result in HBV-specific B cell serum transformation and response impairing, as well as CD8 T cell response defecting (29). PEG-IFNa-2a therapy was associated with an increase of CD56bright cells and distinct changes in expression profiles leading to an activated NK cell phenotype, increased functionality and decline of terminally differentiated NK cells. Ribavirin combination therapy reduced some of the IFN effects. An activated NK cell phenotype during therapy was inversely correlated with HCV viral load (31). Growing evidence shows that adaptive (T and B lymphocytes, as well as monocytes/macrophages and dendritic cells) and innate (γ/δ T cells and natural killer cells) immune responses may be involved in the pathogenesis of hypertension (22). In general, it is widely accepted that the immune system (especially T lymphocytes) is involved in the pathogenesis of hypertension, but the mechanistic processes preceding the activation of immune cells remain unclear. One of the possible underlying mechanisms may be related to sympathetic activity increasing, which is a complex issue because sympathetic excitation may induce both excitatory and inhibition effects of T lymphocytes. Specifically, there is evidence that increased sympathetic outflow may exacerbate inflammatory effects of activated T lymphocytes and also directly attenuate the ability of naive T lymphocytes to become fully activated T lymphocytes (23). Systemic chronic low-grade inflammation is present in type 2 diabetes mellitus (T2DM). Function alteration of specific T lymphocyte subsets (including regulatory T (Treg) cells) results in the hypothesis thatT2D autoimmunity was exacerbated by partial inflammation (30). Our previous study showed that the lowest lymphocyte counts, especially T and B subsets were found in patients with severe COVID-19 and DM (15–18). Previous study showed that obese diabetic subjects presented much higher levels of polyclonal activation and antibody secretion in B cell, with impaired ability to response to new antigens such as seasonal influenza vaccination. Elevated B Cell Activation is Associated with Type 2 Diabetes Development in Obese Subjects (32). Another previous study showed that compared with individuals with normal glucose tolerance or prediabetes, type 2 diabetes patients had a reduced NK cell activity (33). Growing evidence shows that adaptive (T and B lymphocytes, as well as monocytes/macrophages and dendritic cells) and innate (γ/δ T cells and natural killer cells) immune responses may be involved in the pathogenesis of hypertension (22). In NAFLD, liver sentinel cells such as resident Kupffer cells and monocyte derived macrophages respond rapidly to local and persistent increasing exogenous antigens, metabolites, and pattern molecules. Then the liver becomes an immune-active phenotype from an immune-tolerant state which reduces production of anti-inflammatory cytokines such as interleukin-10 (IL10) and transforming growth factor β (TGF β), while increases production of pro-inflammatory cytokines such as tumor necrosis factor α (TNF-α), IL1 and IL6. In turn, in NASH livers the interaction between hepatocytes and adaptive and innate immune cells drives chronic low-grade inflammation (34, 35). Diffuse lymphocyte lobular infiltration is one of the histological features of nonalcoholic steatohepatitis (NASH) (36, 37). B cells and T cells form focal aggregates are found in approximately 60% of NASH patients (38). Liver infiltration of B cells, CD4+, and CD8+ T cells was evident in different NASH models, which exacerbated liver parenchymal injury and lobular inflammation (37, 39, 40). These T cells express the memory or effector markers CD25, CD44 and CD69, along with enhanced production of the cytokine LIGHT, indicating their functional activation (37, 39, 40). It has been widely recognized that immune function declines with age (41). Previous study found that the peripheral blood immune cells of CHB patients showed various disorders and functional impairment, especially NK cells and T cells exhaustion, which was manifested as increased expression of inhibitory receptors such as NKG2A and PD-1 in both NK and T cells, impaired cytokine secretion function, not significant decrease in cytotoxicity, reduced proportion of dendritic cells (DC) and increased proportion of regulatory B cells (Breg) (42). Therefore, specific comorbidities associated with immunodeficiencies (including cancer, CHB, CKD, NAFLD, CVD and DM) often coexist with SARS-CoV-2 infection, which leads to host immune responses dysregulation and thus exacerbating already aggravated inflammatory processes. Patients with COVID-19 are more severe organ damage, more susceptible to bacterial infections and a poor prognosis (15–18).

To our knowledge, this is the first study reporting on comorbidities impacting on cellular immunity of patients with COVID-19. Based on these findings, a high number of comorbidities and some specific comorbidities with immune response disorders, such as cancer, CHB, CKD, NAFLD, CVD and DM, could impact the immune responses in COVID-19 patients.

Our study had limitations, and one limitation is that our study was a single-center study. Despite this limitation, we report several novel findings: a high number of comorbidities and some specific comorbidities with immune response disorders, such as cancer, CHB, CKD, NAFLD, CVD and DM, could impact the immune response of COVID-19 patients.

## Conclusions

A high number of comorbidities and some specific comorbidities with immune response disorders, such as cancer, CHB, CKD, NAFLD, CVD and DM, could impact on immune response of patients with COVID-19. For clinicians, these study findings provide a reference that the early identification regarding the suitability of immunotherapy in COVID-19 patients to help slow disease progression and improve patients’ prognoses.

## Data Availability Statement

The raw data supporting the conclusions of this article will be made available by the authors, without undue reservation.

## Ethics Statement

The studies involving human participants were reviewed and approved by The Ethics Committee of the Public and Health Clinic Centre of Chengdu approved this study (ethics approval number: PJ-K2020-26-01). Written informed consent for participation was not required for this study in accordance with the national legislation and the institutional requirements.

## Author Contributions

Concept and design: DL, XY, FG, BZ, LD, MH, CL, and LJ; Data acquisition: DL, XY, FG, BZ, LD, MH, and CL; Data analysis and interpretation: DL, XY, FG, BZ, LD, MH, and CL; Drafting the manuscript: DL, XY, FG, BZ, LD, MH, and CL; administrative, technical, or material support: DL, XY, FG, BZ, LD, MH, CL, and LJ; study supervision: LJ. All authors contributed to the article and approved the submitted version.

## Funding

This research was supported by the Nonprofit Central Research Institute Fund of the Chinese Academy of Medical Sciences (2020-PT330-005), the Sichuan Science and Technology Program (2020YFS0564), the Chengdu Municipal Health Commission (2019079), and the Chengdu Science and Technology Bureau (2021-YF05-00536-SN).

## Conflict of Interest

The authors declare that the research was conducted in the absence of any commercial or financial relationships that could be construed as a potential conflict of interest.

## Publisher’s Note

All claims expressed in this article are solely those of the authors and do not necessarily represent those of their affiliated organizations, or those of the publisher, the editors and the reviewers. Any product that may be evaluated in this article, or claim that may be made by its manufacturer, is not guaranteed or endorsed by the publisher.
